# From Phenomenology to Neurophysiological Understanding of Hallucinations in Children and Adolescents

**DOI:** 10.1093/schbul/sbu029

**Published:** 2014-06-13

**Authors:** Renaud Jardri, Agna A. Bartels-Velthuis, Martin Debbané, Jack A. Jenner, Ian Kelleher, Yves Dauvilliers, Giuseppe Plazzi, Morgane Demeulemeester, Christopher N. David, Judith Rapoport, Dries Dobbelaere, Sandra Escher, Charles Fernyhough

**Affiliations:** ^1^Child & Adolescent Psychiatry Department,University Medical Centre Lille,Lille,France;; ^2^Lille Nord de France University, UDSL, Functional Neurosciences & Disorders Lab, Lille, France;; ^3^University of Groningen, University Medical Center Groningen, University Center for Psychiatry, Groningen, The Netherlands;; ^4^Adolescence Clinical Psychology Research Unit, Faculty of Psychology and Educational Sciences, University of Geneva, Geneva, Switzerland;; ^5^Research Department of Clinical, Educational and Health Psychology, University College London, London, UK;; ^6^Jenner Consult Haren & Audito, Practice for Child & Adolescent Voice Hearers, Ten Boer, The Netherlands;; ^7^National Centre for Suicide Research and Prevention of Mental Ill-Health, Karolinska Institutet, Stockholm, Sweden;; ^8^Sleep unit, Department of Neurology, Hôpital Gui-de-Chauliac, CHU Montpellier & National Reference Network for Narcolepsy, INSERM U1061, Montpellier, France;; ^9^Department of Biomedical and Neuromotor Sciences, University of Bologna, Bologna, Italy;; ^10^IRCCS, Institute of Neurological Sciences, Bologna, Italy;; ^11^Lautréamont Clinic, ORPEACLINEA Group, Loos, France;; ^12^Child Psychiatry Branch, National Institute of Mental Health, Bethesda, MD;; ^13^University of Pittsburgh School of Medicine, Pittsburgh, PA;; ^14^National Reference Center for Inherited Metabolic Diseases in Child and Adulthood, University Children’s Hospital Jeanne de Flandre, Lille, France;; ^15^City University of Birmingham, Birmingham, UK;; ^16^Department of Psychology, Durham University, Durham, UK

**Keywords:** hallucinations, childhood, review, adolescence

## Abstract

Typically reported as vivid, multisensory experiences which may spontaneously resolve, hallucinations are present at high rates during childhood. The risk of associated psychopathology is a major cause of concern. On the one hand, the risk of developing further delusional ideation has been shown to be reduced by better theory of mind skills. On the other hand, ideas of reference, passivity phenomena, and misidentification syndrome have been shown to increase the risk of self-injury or heteroaggressive behaviors. Cognitive psychology and brain-imaging studies have advanced our knowledge of the mechanisms underlying these early-onset hallucinations. Notably, specific functional impairments have been associated with certain phenomenological characteristics of hallucinations in youths, including intrusiveness and the sense of reality. In this review, we provide an update of associated epidemiological and phenomenological factors (including sociocultural context, social adversity, and genetics, considered in relation to the psychosis continuum hypothesis), cognitive models, and neurophysiological findings concerning hallucinations in children and adolescents. Key issues that have interfered with progress are considered and recommendations for future studies are provided.

## Introduction

Seeing, hearing, touching, smelling, or tasting constitute some of the earliest experiences of life, to which may be added experiences of internal origin such as emotions. The sensory systems involved can be functionally evidenced during prenatal life, and the resulting experiences help the growing child to build representations of the surrounding world and progressively access higher consciousness. Perception not only involves bottom-up sensory processing but also inferential, top-down processes, leading to the optimal combination of new sensory inputs with prior knowledge. In this sense, recent neuroscientific progress converges with philosophical views stating that the physical world is only partially accessible to the subject and is always accessed through a strongly subjective prism.

Considering this framework, how do children apprehend their sensory environment, particularly before the development of the meta-representational skills purported to be necessary in distinguishing reality from fantasy? At what age may hallucinations, defined as erroneous percepts in the absence of identifiable stimuli, initially be observed? Do they occur in drowsiness, wakefulness, or in more than one state? Are all of these experiences pathological, particularly when considered in the context of typical child development?

When defined according to the above definition, hallucinations are relatively common in the pediatric population. For example, considering psychotic experiences generally (including hallucinations), Laurens et al^1^ estimated that two-thirds of children 9–11 years old (y.o.) had experienced at least one psychotic-like experience.^1^ More specifically, auditory-verbal hallucinations (AVH) but also visual, tactile, or gustatory hallucinations can be frequently observed in clinical and nonclinical pediatric populations.^2^ The purpose of this article is to systematically review studies on childhood and adolescent hallucinations and attempt to synthesize the main results, emphasize the problems that have interfered with progress, and make recommendations for future research.

## The Epidemiology and Phenomenology of Hallucinations in Children and Adolescents

### Hallucinations in the General Pediatric Population

Studies of large pediatric samples documented a 8% hallucination prevalence rate in children,^3^ which substantiates previous estimates of 5% in 5–15 y.o. children and adolescents.^4^ One of the lowest population estimates of nonpsychotic subjects with hallucinations was 1.1%, although the study sample size could be considered relatively small (*n* = 40).^5^ One of the highest prevalence rates was observed in a nonclinical sample, in which 21.3% of the 761 11–12 y.o. Japanese children enrolled had experienced visual and/or auditory hallucinations.^6^ This highlights the fact that sociopsychological factors, such as cultural or religious differences, must be considered when comparing studies of prevalence (see also “Cultural and Spiritual Factors in Early-Onset Hallucinations” section).

Furthermore, looking at hallucinatory experiences in 337 children aged 7–8 years at baseline that were reassessed after a mean follow-up period of 5.1 years, the cumulative incidence rate for AVH in the baseline sample was estimated at 9%.^7^ Importantly, the vast majority of hallucinations in the general pediatric population are transient and resolve spontaneously. In approximately 50%–95% of cases, hallucinations discontinue after a few weeks or months.^8^ For some young people, most nonpsychotic hallucinations are associated with periods of anxiety and stressful events and disappear when the stressful situation is resolved.^9^

### Imaginary Companions

Clinical assessments in children are further complicated by the high rate of immature responses that, on some definitions, fit the criteria for hallucinations. Among such phenomena are imaginary companions, which could be described as “*hallucination-like phenomena*.”^10,11^ Twenty-eight percent to 65% of school-age children (ie, between the ages of 5–12 y.o.) report such experiences.^11^ On the one hand, a positive correlation was observed between the presence of a parentally corroborated imaginary companion and the tendency to hear words when the child was placed in a noisy or ambiguous testing environment.^10^ On the other hand, the presence of imaginary companions has been associated with positive developmental outcomes, such as theory of mind (ToM) performance, one of several reasons for thinking that imaginary companions should not be considered markers of psychosis^12^ (also see “Functional Brain Imaging of Early-Onset Hallucinations” section).

From a clinical perspective, these latter experiences differ from hallucinations in at least 2 respects: (1) they can often be invoked by the child at will in contrast with the involuntary nature of hallucinations (although so-called “*noncompliant imaginary companions*” are resistant to the host child’s control^13^) and (2) they typically function as playing partners associated with positive emotions (again, with the exception of some noncompliant companions; see also table 2).

### Sleep-Related Hallucinations in Children and the Specific Case of Narcolepsy

Other phenomena observed during the developmental period include hypnagogic and hypnopompic hallucinations, which occur immediately before falling asleep and during the transition from sleep to wakefulness, respectively. Both these experiences are believed to represent the same group of phenomena, reported in 25% and 18% of the general population respectively,^14^ and observed to decline with age in adulthood. Besides normal-range experiences, there are specific cases in which sleep-related hallucinations may be part of a disabling childhood sleep disorder. For example, narcolepsy with cataplexy (NC) is characterized by a specific dysregulation of the sleep-wake cycle, with an increased penetration of rapid eye movement (REM) sleep.^15^

NC combines excessive daytime sleepiness, cataplexy, and sleep paralysis with hypnagogic or hypnopompic hallucinations. The prevalence of NC is 0.026% with the main peak of disease onset at 16 years.^15^ Sleep-related hallucinations occur in approximately 50%–70% of NC patients with a mean onset age of 10.4 (± 3.5 years)^16^ and are often described as vivid, frightening and mainly occurring in the auditory (phone ringing), visual (threatening figure, animals, or person), or somesthetic (out-of-body experience) modalities. NC patients often present multisensory hallucinations, but AVH are rare.^17^ Hallucinations may occasionally be so frightening that patients with NC become fearful of going to bed. As with early-onset hallucinations in general, many individuals with narcolepsy are unwilling to talk about these symptoms. The presence of excessive daytime sleepiness, cataplexy, and atypical automatic behaviors may orient the clinician,^18^ whereas a polysomnography assessment showing abnormal REM sleep architecture, the presence of HLA DQB1*0602 and undetectable cerebrospinal fluid-hypocretin-1 levels may further guide the differential diagnosis.^15^

### Psychopathology and Early-Onset Hallucinations

Mood disorders can often present with accompanying psychotic features, including hallucinations.^19^ Significant incidence rates of hallucinations (up to 37% are pathological when combined with delusions) have been observed in child bipolar type-I disorder.^20^ Research has also documented cases of nonpsychotic children who hallucinate and have diagnoses of attention deficit hyperactivity disorder (ADHD) (22%), major depressive disorder (MDD, 34%), disruptive behavioral disorders (21%), and other diagnoses (23%).^21^ Furthermore, studies have reported that pediatric patients with Tourette’s syndrome associated with obsessive-compulsive disorder or ADHD also report a higher rate of auditory and visual hallucinations.^22^

A recent multicenter study of 11–16 y.o. adolescents^23^ found that a majority of young people in the population who reported hallucinations had at least one lifetime mental disorder. Hallucinations were associated with a wide range of disorders but were in particular strong markers of risk for multimorbid psychopathology, ie, the presence of more than one psychiatric diagnosis. The prevalence of psychotic experiences increased in a dose-response manner in line with the number of diagnosable psychiatric disorders. This has also been demonstrated in clinical populations, with 11–15 y.o. patients who reported psychotic experiences were found to have, on average, 3 diagnosable DSM IV (Diagnostic and Statistical Manual of Mental Disorders, 4th edition) Axis-1 disorders.^24^ Additionally, psychotic symptoms predicted more severe psychopathology from a number of perspectives in addition to multimorbidity, in terms of both global and cognitive functioning.^25^

A significant relationship between hallucinations and suicidal behavior has also been demonstrated.^26^ In a community-based study of adolescents, those with psychiatric disorders who reported psychotic experiences (predominantly AVH) had a far higher prevalence of suicidal behavior than those with psychopathology who did not report psychotic experiences.^25^ Adolescents with a diagnosis of MDD who reported psychotic experiences, eg, had a 14-fold increase in suicide plans or attempts compared to adolescents with the same diagnosis who did not report psychotic experiences. In a prospective cohort study, AVH were also investigated as a clinical predictor of future suicide attempts in community-based adolescents.^26^ By 1-year follow-up, more than one-third of adolescents with baseline psychopathology who reported AVH had a suicide attempt, compared to 13% with baseline psychopathology who did not report AVH.

### Childhood-Onset Schizophrenia

Childhood-onset schizophrenia (COS) constitutes a rare subset of cases typically occurring before 13 y.o. (prevalence ~1/30 000). Despite its rarity, recent studies have shown that schizophrenia can be reliably diagnosed in children and that it is neurobiologically, diagnostically, and physiologically continuous with the adult disorder.^27,28^ Work with the National Institute of Mental Health (NIMH) COS cohort has accrued 2 decades of clinical rating data and a recent report has documented high rates of hallucinations across all sensory modalities.^29^ Nearly all COS patients described significant auditory hallucinations. More importantly, a high rate of the cohort reported significant rates of visual hallucinations (80%) during their hospitalizations.^29^ High rates for tactile (60%) and olfactory (30%) hallucinations were observed in this pediatric sample, which occurred exclusively in patients with visual hallucinations.

These high incidence rates may reflect the severe psychopathology of the NIMH COS inpatient population (*n* = 117). Visual hallucinations showed a significant association with lower IQ and earlier age of psychosis onset.^29^ In particular, verbal IQ demonstrated a consistent inverse relationship with the presence of visual, somatic/tactile, and olfactory hallucinations, even when each modality was considered independently, and these modalities thus appear to be a general marker of an increased severity of psychosis. However, it is notable that COS is extremely rare, and the majority of children experiencing hallucinations do not progress to COS. The dramatic results from the NIMH COS sample are atypical in terms of the early age of onset and antipsychotic medication resistance, and therefore may not generalize to other samples of adolescents with hallucinations.

### Persistence of Hallucinations and Predictive Value During Development

Age may modulate the relationship between hallucinations and psychopathology. Hallucinations were shown to be only associated with a minor increase in risk for psychiatric symptoms in 7–8 y.o. children,^30^ but predicted an approximate 3- to 5-fold increase in the odds of scoring within the clinical range of the Child Behavior Checklist (CBCL) at 12 and 13 y.o. (notably, this increase was highly significant for the “somatic complaints” CBCL syndrome subscore). Persistence of childhood hallucinations during adolescence may occur in 23.5%–27%.^7,31^ The specific risk of developing psychosis remains present with an estimated 5- to 6-fold higher risk when hallucinations persist during adolescence.^32,33^ The factors most often associated with persistent hallucinations are frequency, the number of voices, negative tone, and presence of passivity phenomena, comorbidities, and a lower level of global functioning. Furthermore, cannabis exposure has been suggested to increase hallucination persistence.^34^

A growing body of evidence suggests that psychotic-like experiences that encompass delusionary or hallucinatory experiences occur much more frequently in the general population than do psychotic disorders, suggesting a symptomatic continuum within the community.^35^ The Persistence-Impairment (P-I) model has recently been associated with a similar phenomenological continuity between clinical and subclinical psychosis phenotypes.^36^ The P-I model implies a probabilistic relationship (following a dose-response relationship), in which risk factors (eg, urbanicity, trauma, migration, ethnic minority status, substance misuse) increase stress or reduce the potential compensatory processes, which consequently increase hallucinatory experiences for both constituted psychotic disorders and isolated symptoms in the general population.^37,38^

This theory is supported by the following sources: (1) epidemiological studies that reveal an increased number of dynamic transitions over time from subclinical manifestations to full-blown psychotic disorders as a function of the additional risk factors,^32,39^ (2) comorbidity studies that reveal a linear relationship between early-onset hallucinations and the number of diagnosable psychiatric disorders,^23^ and (3) cognitive studies that reveal modest impairments in mentalizing, verbal memory, executive functioning, and attention in the nonclinical relatives of patients with psychosis^40^ or in patients with schizotypal personality disorders^41^ (see “Cognitive Theories of Hallucinations in Children and Adolescents” section; see also Johns et al, this issue).

Overall, these studies show that the presence of isolated hallucinations in childhood is insufficient to predict clinical outcomes and needs to be considered against the environmental, phenomenological, psychological, sleep/wake occurrence, developmental, and sociocultural contexts in which an individual has these experiences. Hallucinations often fall within the normal spectrum of experience in childhood but may be expected to discontinue in the course of normal healthy development.^10^ Hallucinations that occur or persist during adolescence become increasingly associated with psychopathology, in particular with severe, multimorbid diagnoses associated with poor functioning and risk for suicidal behavior.

## Factors Associated With Hallucinations in Children and Adolescents

### Cultural and Spiritual Factors in Early-Onset Hallucinations

The reference to a more ecological framework also constitutes a major shift in the way hallucinations can be investigated during childhood. Notably, it has been reported that in non-Western cultures, altered states of perception, such as hallucinations, were more frequently attributed to possession by spirits or an attempt to establish contact with spirits^42^ (see also Larøi et al, this issue). Overall, the occurrence rate, the sensory modalities involved, and the attribution styles were cited as the most common phenomenological changes in early-onset hallucinations that may be affected by culture. A national community survey conducted in the United Kingdom, eg, revealed significant variations in hallucination reports across ethnic groups, most notably among 16–19 y.o., with the highest rates in the Caribbean group (9.8%) and the lowest in the South-Asian group (2.3%).^43^ An increased rate of visual hallucinations (17.4%) was also observed in a Kenyan adolescent and preadolescent community sample compared with other psychotic-like experiences, such as paranoia and referential thinking.^44^

A special emphasis has also been placed on the impact of migration, as cited, eg, in a recent World Health Organization study revealing an increased prevalence of psychotic disorders in migrants, which persisted in the second and third generations after adjusting for age, sex, level of education, and cannabis use.^45^ However, the true influence of culture and origin on the phenomenology of hallucinations was also put into question by drawing a comparison with the role played by the immediate environment.^46,47^ Within the full sociocultural background, magically oriented folk beliefs and religious practices may affect secondary delusional content, even if these factors do not directly lead to higher rates of religious hallucinations or noncompliance with psychiatric care.^48^ Even if these shortcomings persist, most notably due to the lack of a valid cross-cultural definition of hallucinatory experiences, clinicians need to consider the fact that many children are reared in a dual system of public and private cultures according to the heritage of their specific ethnic group,^49^ indicating the need to assess their spiritual or magical beliefs.^50^

### Early Trauma and Hallucinations

Several studies have demonstrated that experiencing childhood trauma is a risk factor for psychosis. A recent meta-analysis yielded odds ratios between 2.38 and 3.40 of developing psychosis as a result of sexual abuse, physical abuse, emotional abuse, bullying, or neglect, but not parental death.^51^ Besides childhood traumatic experiences, other indicators of social adversity, such as stressful life events, may elicit psychotic experiences.^52^ In addition to consistent evidence of this relationship, Bentall and colleagues^53^ also observed a dose-response effect of childhood adversity and AVH.

A 3-year prospective follow-up study by Escher et al^52^ of 80 voice-hearing individuals aged 8–19 years revealed that childhood adversity was observed in a small group of children, whereas high scores on life events relating to the onset of hearing voices were observed in the entire group.^52^ Of the children, 86.3% reported one or more traumatic events around the time of voice onset. Bartels-Velthuis and colleagues^54^ reassessed 337 children (mean age 13.1 years, range 12.0–14.6), pertaining to a baseline case-control sample (*n* = 694) of 7 and 8 y.o. from a general population with and without AVH. These authors also observed a significant association of the social adversity level (both traumatic and stressful events were assessed) and hearing voices. In addition, adolescents with a combination of AVH and delusions experienced significantly more traumatic and stressful events than those with either AVH or delusions alone.

The mechanism that underlies the complex associations between clinical psychotic symptoms and childhood trauma may represent the persistence of initially subclinical psychotic experiences and complicate initial hallucinatory experiences by (secondary) delusional ideation.^55^ Children with AVH who experience traumatic and stressful events may develop delusional ideation, but a reverse causality was also considered by some authors.^56^ For instance, children previously showing subtle alterations in behavior or unusual ideas might be more susceptible to bullying by peers or be otherwise victimized, thus experiencing more adversity.^57,58^ Recent research, in fact, has demonstrated that there is a bidirectional relationship between childhood trauma and psychotic experiences, with each independently predicting the other.^59^

### Genetic Factors and Early-Onset Hallucinations

The specific heritability of hallucinations was recently addressed in large twin samples.^60,61^ Additive genetic effects were observed to account for 33% (95% CI: 23%–42%) of the variance of the hallucination score in 598 twin pairs without gender effects. This result was strikingly below the heritability scores in twin pairs examined for thought disturbance via parent reports, which reached approximately 75%. These discrepancies suggest that heritability studies should specifically assess hallucinations and preferably rely on self-reports or semistructured interviews with children or adolescents rather than parental or external informants.

Studies conducted in adults have implicated 3 main candidate genes in different facets of AVH: the serotonin transporter gene (*5-HTT*), the cholecystokinin receptor gene (*CCKAR*), and the forkhead box protein P2 gene (*FOXP2*). Other clues to the genetic basis of hallucinations have been provided by studies on the GABRB3 191bp allele in patients with schizophrenia and AVH, and examination of individuals with 22q11.2 deletion syndrome who may experience transient hallucinatory phenomena during adolescence. Because hallucinatory experiences most often involve significant emotional experiences, the serotoninergic system has been proposed to modulate the emotional reactions linked to AVH. While the *5-HTT* long allele (L), associated with increased transcriptional activity of the gene—ie, responsible for increasing the recapture of serotonin in the synaptic cleft—has been associated with AVH intensity,^62^ a second study demonstrated that the implication of the short allele (S) of the *5-HTT* gene was specifically linked with increased emotional reactions and emotional distress in reaction to AVH.^63^

The *CCK* *AR* gene, which codes for receptors of cholecystokinin, has been previously suggested to sustain schizophrenia pathogenesis by mediating the availability of mesolimbic dopamine, although it has recently been found to be associated with AVH beyond the schizophrenia spectrum.^64^ Further evidence for the genetic basis of hallucinations can be observed in relation to genes linked to different dimensions of language and audition. The *FOXP2* gene is a natural candidate because of its associations with language disorders and the neural language system. Specific polymorphisms, mainly located in the regulatory region of the gene, are associated with the frequency and duration of hallucinations in schizophrenia patients.^65^ Finally, gene × environment interactions were recently demonstrated through a significant link between specific FOXP2 genotypes, a history of childhood parental emotional abuse, and AVH experiences.^66^

## Cognitive Theories of Hallucinations in Children and Adolescents

### Cognitive Profiles and Early-Onset Hallucinations

Studies obtaining cognitive profiles of children with hallucinations have been valuable in providing clues to underlying cognitive mechanisms. By comparing healthy adult individuals with and without AVH, Daalman et al^67^ showed that individuals with AVH were more sensitive to distraction (lower performance on inhibition), had a lower verbal working memory capacity (lower digit-span backward performance), and underperformed on Wechsler Adult Intelligence Scale III subtests of vocabulary and similarities. These authors postulated that a specific alteration in the executive functioning combined with a reduced level of verbal IQ might increase the tendency to hallucinate in the auditory-verbal domain, explicable in terms of difficulties in the inhibition of irrelevant verbal information. Another study of 11–13 y.o. participants confirmed lower performance on executive functions (mental flexibility and verbal abstraction) and language (denomination of images) in hallucinators compared with healthy subjects,^68^ along with impairments of fine motor skills and information processing speeds. These latter elements were additionally shown to be risk factors for psychosis.^69^

### ToM in Children With Hallucinations

ToM is defined here as the ability to correctly interpret another individual’s intentions or emotions. Several studies have revealed that patients with schizophrenia,^70–73^ as well as nonclinical adults with schizotypal traits, exhibit impairments in ToM^74^ (although, see eg, Fernyhough et al^75^). Few studies have focused on the direct relationship between AVH and ToM. Although Abdel-Hamid and colleagues^76^ demonstrated weaker ToM abilities in schizophrenia patients, they did not establish a significant association between ToM deficits and positive symptoms, such as hallucinations. Delusions may be secondary to abnormal perceptual processes^77^ and aberrant attributions of salience.^78^ The pathway from AVH to delusions might therefore be explained by a misinterpretation of these percepts, raising the questions of whether and how (social) cognitive processes may be involved. As Smeets et al^55^ observed, the combination of hallucinations and delusions, rather than either one in isolation, may contribute to a persistence of symptoms and a deterioration in clinical outcomes. The results of Smeets and colleagues were based on data collected at 2 time points from the Early Developmental Stages of Psychopathology (EDSP) Study in a large representative general population sample of adolescents and young adults (mean ages 21.8 and 26.6 years, respectively).

In children, associations between ToM and AVH, and the role of delusion formation, have also been examined. In a prospective longitudinal birth cohort study in Great Britain (following 2232 children), it was demonstrated that 12 y.o. children with psychotic symptoms (including AVH) exhibited impaired ToM at age 5.^79^ A negative appraisal of the tone of the voices heard in children was also proposed to elicit delusion formation,^52^ but an absence of reasoning biases was shown able to prevent the formation of threatening appraisals about anomalous perceptions.^80^ Finally, a study by Bartels-Velthuis and colleagues^81^ focused on the role of ToM in the process of delusion formation in 12 and 13 y.o. preadolescents with AVH. The authors suggested that better mentalizing abilities might confer protection against delusion formation in children experiencing perceptual anomalies, an effect that was not reducible to general cognitive ability.

### Functional Brain Imaging of Early-Onset Hallucinations

Progress in brain-imaging technology has paved the way for the noninvasive exploration of the neural structures involved in hallucinations^82^ during development. Two main categories of brain-imaging studies are conceptually available on the problem of early-onset hallucinations. Initially, studies compared hallucinators with non-hallucinators. These “trait studies” investigated the neural bases of the susceptibility to hallucinate, because no information on the child/adolescent experience during scanning was available. Comparing 11–13 y.o. preadolescents with psychotic-like experiences with matched healthy controls, an initial set of experiments showed functional changes encompassing the error-related processing network,^83^ the perspective-taking network,^84^ and the status of intrinsic functional connectivity within the inhibitory control network,^85^ in line with behavioral findings previously mentioned (see “ToM in Children With Hallucinations” section). Such differences measured in the blood-oxygen-level-dependent (BOLD) signal—during cognitive tasks and at rest—were shown to be associated with gray matter^83,86^ and event-related potentials changes^87^ in at-risk or first-episode adolescents compared with controls, supporting a hypothesis of distributed neural impairments associated with the psychosis phenotype in general.

The second category of experiments is “state studies,” ie, studies conducted during the occurrence of hallucinations.^88^ State studies allow researchers to directly measure brain activations associated with symptom emergence. A recent multimodal magnetic resonance imaging (MRI) experiment conducted on 20 11–16 y.o. adolescents with brief psychotic episodes and suffering from auditory, visual, and multisensory hallucinations confirmed an increase in the BOLD signal within modality-dependent association sensory cortices during hallucinatory experiences.^89^ Notably, recruitment of primary sensory cortices was not systematic and was observed, when present, to be associated with increased, vivid hallucinatory experiences. By exploring the neurodynamic patterns of the default-mode network (DMN) and associative sensory cortices during hallucinations, these authors showed spatial and temporal instabilities of the DMN that correlated with severity of hallucinations and persisted during symptom-free periods,^89^ suggesting an intrinsic instability of the DMN in this population compatible with the phenomenological properties of these experiences, specifically their intermittent nature.

### Neuromodulation and Early-Onset Hallucinations

In neuroscience, transcranial magnetic stimulation (TMS) has been used as a complementary technique to brain imaging in cognitive mapping. This method has been especially useful for exploring the neural bases of self-other distinctions. For example, sense of agency and the severity of hallucinations was shown to be functionally dissociable in a functional MRI (fMRI)-guided TMS case report that specifically explored these functions in an 11 y.o. child with COS.^90^ Few studies have examined the neurocognitive bases of early-onset hallucinations, indicating that replications in larger samples of children or adolescents are required.

TMS was also proposed as a second-line therapeutic strategy for refractory AVH occurring in children and adolescents. By focally modulating neural excitability, these methods allow for a dimensional and noninvasive add-on therapy for hallucinations. In adults, repetitive TMS (rTMS) centered on the left temporal-parietal junction has been shown to be effective in reducing the severity of AVH. A recent meta-analysis confirmed a 0.42 effect size for rTMS in this indication, which constitutes a moderate but significant effect when compared with placebo.^91^ rTMS has shown good tolerance in pediatric populations,^92^ as was shown by recent preliminary data on the effectiveness of rTMS in relieving early-onset hallucinations. Decreased symptom severity and improved global functioning were shown immediately after treatment and at subsequent reassessment 1 month later in a cohort of 10 adolescents.^93^ Although these results appear encouraging, replication in larger groups, looking at long-term effects, and according to evidence-based standards (ie, randomized control trial designs) is now required.

## Clinical Considerations

Although a systematic description of organic disorders associated with early-onset hallucinations extends beyond the scope of the present review, readers should remain aware that these experiences may also occur in the context of pediatric neurological disorders (migraines with aura, complex partial seizures, malignancies, or brain lesions, among others), infections, autoimmune disorders, or metabolic disorders. Indeed, as previously mentioned regarding the P-I model for psychotic experiences, whatever the underlying mechanism of the stressors (ie, psychosocial or biological), they increase the probability that hallucinations are clinically significant and require care. The observation of hallucinations in children and adolescents therefore requires a clinical examination and psychological assessment to rule out medical causes and identify the psychopathological, psychosocial, and cultural factors associated with these experiences (tables 1 and 2).

**Table 1.** The Diagnostic Steps to Assess Hallucinations in Children and Adolescents. Adapted From Jenner JA, *HITting Voices* (submitted)

a: Probe experiences, physical qualities distinct from the illusions, and obsessive-compulsive thoughts.
b: Assess danger (suicidality, the presence of misidentification syndrome, etc.).
c: Assess somatic and psychiatric disorders, drug abuse, and medication. Administer physical examination. The timing and comprehensiveness of complementary exploration are debatable. Second-line tests may be offered depending on the clinical examination (eg, blood count and urine toxicology are usually recommended. A genetic consultation will only be proposed in the presence of dysmorphologies, intellectual disability, or congenital malformations and will be used to help decide whether microarray testing is necessary).
d: Habitual reaction patterns and personality traits indicate the preferred style of therapy.
e: Assess hallucination characteristics (eg, frequency, intensity, conviction, insight, degree of control, discomfort in daily life, distress, emotional valence, coping strategy), their sense and meaning in relation with sociocultural factors, and the patient’s and relatives’ explanations using standardized instruments.
f: Assess functionality, secondary gain, and reinforcement.
g: Probe into habitual coping behavior.
h: Estimate compliance and the balance between unwillingness and incapacity.

Few psychometric tools have been specifically developed to assess early-onset hallucinations accounting for both progressive cognitive development and comprehension levels in children and adolescents.^94–96^ Both social cognition and delusion formation should be assessed in young individuals with AVH that are seeking help, in order to provide tailored care aimed at decreasing the risk of a possible poor prognosis. To our knowledge, there is no evidence-based scheme available to reach a therapeutic decision based on optimized clinical and second-line tests. Diagnostic steps must precede treatment (table 1), and choosing the best program will depend on age, severity, burden, and comorbid disorders (table 2).

**Table 2.** Stepwise Approach for the Treatment of Early-Onset Hallucinations

1. Reference to the developmental context: unusual experiences may be a normal aspect of development (imaginary companions, hypnagogic/hypnopompic hallucinations) or related to cognitive immaturity (preschooler children may apply illogical thinking and describe their thoughts as “voices”). Rather than defining an age frame during which hallucination-like experiences can be considered as part of normal development, clinicians may consider these experiences as nonpathological when associated with the following features: (1) the possibility to be invoked at will by the child, (2) overall positive emotions, and (3) an absence of interference with peer socialization.
2. Destigmatization: the use of the “psychotic” terminology to describe these experiences has been questioned. A preference for “voice hearing,” “visions,” or “hearing or seeing things that other people cannot hear or see” may be less stigmatizing and should be considered.
3. A complete assessment: considering the characteristics of both the hallucinations and of associated disorders is mandatory, and addressing the risk factors is warranted.
4. Normalization, support, and reassurance may be sufficient in most hallucinating children. Involving the family (especially parents) is recommended.
5. If treatment is indicated, giving the hallucinations a pivotal role may increase compliance without neglecting therapeutic actions centered on identified associated factors (delusional ideation, bullying, emotional coping capabilities, stress).
6. Hallucination-focused therapies that are also cause-oriented should be considered.

Destigmatization, normalization, and reassurance may offer the best course of action in the absence of pathological signs. The presence of suicidality, substance use, and pathological scores on the CBCL, as well as psychiatric, somatic, or psychosocial comorbid disorder(s), may require specific interventions that diverge from the disorder-oriented therapies. Cognitive behavior therapy^97^ and hallucination-focused integrative therapy^98,99^ are evidence-based interventions. Specific interventions centered on ToM are advisable (see also “ToM in Children With Hallucinations” section), while etiological considerations must be privileged when possible (eg, selective serotoninergic recapture inhibitors for MDD, antipsychotic medication for COS, or sodium oxybate for cataplexy). In children with multiple medications, the dosage and number of medications should be carefully reviewed and, if possible, reduced, before adding a new medication. Finally, beyond evidence for the concepts of feasibility and efficacy, the exact placement of new techniques, such as rTMS, in the therapeutic repertoire for child psychiatrists facing early-onset hallucinations must be defined. Future goals also include tailored therapy based on individualized fMRI data.^100^

## Conclusion and Future Directions for Research

It is commonly considered that hallucinations are difficult to assess in children before the acquisition of good language skills. The phenomena may be transitory or a benign consequence of children’s magical beliefs. Sleep-related events also need to be considered. However, hallucinations have shown to be intimately linked with psychopathology, particularly when occurring in adolescence, and may occur in the context of early traumatic events or in association with a depressive disorder. Caution is required before diagnosing COS in children with hallucinations, but associations with negative prodromal symptoms (eg, isolation and psychomotor poverty) must alert the clinician. Furthermore, AVH, pre- and perinatal risk factors,^101^ and delayed developmental milestones^102^ may be associated with later psychotic disorders. Therefore, examination of the developmental correlates of AVH may clarify the possible pathway to psychosis for which early detection is crucial to reduce long-term disabilities and promote recovery.

**Fig. 1.**
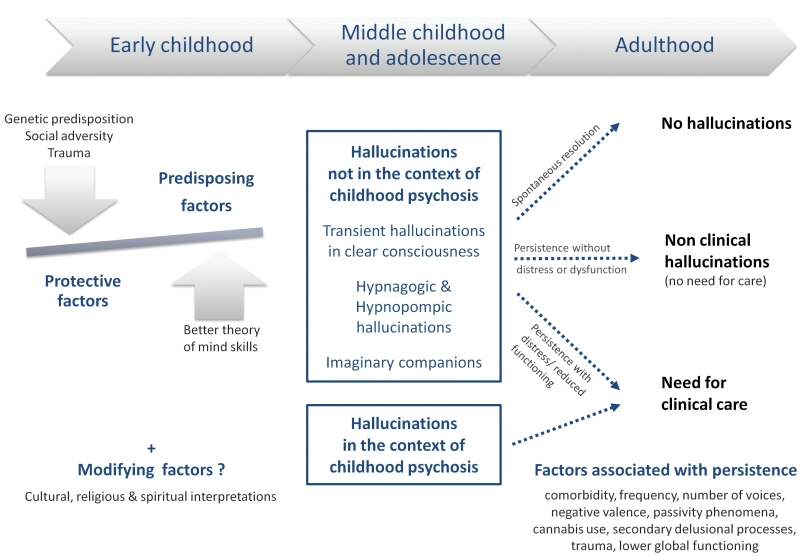
Factors affecting hallucinations in childhood and adolescence. Even if early-onset hallucinations cannot always be totally separated in characteristic profile, trajectory, and outcomes, 2 types of experiences can be distinguished: hallucinations that occur in the context of childhood psychosis and those that do not. Three main categories of factors can then be defined. A first subset of factors influence the occurrence of hallucinatory experiences during childhood (predisposing or protecting factors). Modifying factors (cultural, religious, and spiritual) will affect both the prevalence and the phenomenology of early-onset hallucinations. A third category of factors is associated with persistence of hallucinations during adolescence, and with poorer outcomes. Importantly, based on the extant literature, arrows should be taken to indicate developmental associations rather than causality; in cases where causality might be inferred, causal relations might be unidirectional or bidirectional.

Although early-onset hallucinations may constitute one feature of psychotic experiences, the current literature emphasizes that hallucinations may also occur as isolated phenomena in otherwise healthy children. Uniting the available strands of evidence generates the hypothesis that not only the mere occurrence of a hallucination but also the persistence of hallucinations over time, and particularly into adolescence, may predispose an individual to the secondary formation of delusions and affective dysregulation (cf, figure 1). In this regard, the continuum hypothesis of hallucinatory experiences, although not regarded as the dominant framework of causation for hallucinations, appears to be less stigmatizing relative to conventional categorical approaches (although see Johns et al, this issue for discussion of the possibility of multiple continua for psychosis). Based on the above-outlined issues, practical recommendations for future research are proposed in table 3.

**Table 3.** Practical Recommendations for Future Research

More knowledge is needed regarding the following issues:
1. Building a consensus (in the context of existing discourses around the continuum hypothesis; see Johns et al, this issue) on what should be considered as early-onset hallucinations, clearly distinguishing hallucinations from developmental hallucination-like experiences and simply hearing “noise,” and assessing the phenomenological impact of sociocultural factors.
2. The longitudinal pattern of development in the case of early-onset hallucinations. A large number of available data on early-onset hallucinations rely on cross-sectional or retrospective designs more exposed to methodological bias than longitudinal designs. Epidemiological studies that focus on early-onset hallucinations (and not just psychotic-like experiences in general) may follow these cohorts into adulthood to study the long-term course of hallucinatory experiences and their possible relationship with behavioral problems and to precisely determine the predictive value of hallucinations for later psychiatric disorders (including but not limited to psychosis).
3. Why psychiatric disorders develop when early-onset hallucinations persist? This issue is noteworthy considering the development of translational models integrating biological (eg, genetic) and psychological factors (eg, reduced ToM skills). Studies, particularly focusing on underlying cognitive mechanisms of early-onset hallucinations and their phenomenological quality, should be conducted transdiagnostically to compare hallucinations in children who are healthy, children who have depressive disorders, and children who have COS, among others.
4. New ways to assess hallucinations in young persons based on the increasing development of apps for digital devices (eg, smartphones), constituting intermediate tools between self- and interviewer-based assessments of these experiences. For tools exploring psychotic experiences rather than hallucinations specifically, factor analysis of “perceptual abnormalities” is recommended (ie, using the CAPE).^ 93 ^
5. Therapeutic strategies: Randomized control trials comparing the respective efficacy of the therapeutic strategies available for children and adolescents with hallucinations are necessary to provide clear guidelines to clinicians and should range from psychotherapy to pharmacotherapy and neuromodulation. Crucially, future interventions should be specifically designed for pediatric populations and not just transfer adult-based interventions to young persons.

*Note:* COS, childhood-onset schizophrenia; CAPE, Community Assessment of Psychic Experiences; ToM, theory of mind.

## Funding

Wellcome Trust grant (WT098455 to C.F.).
